# Developmental changes in fronto-striatal glutamate and their association with functioning during inhibitory control in autism spectrum disorder and obsessive compulsive disorder

**DOI:** 10.1016/j.nicl.2021.102622

**Published:** 2021-03-10

**Authors:** Viola Hollestein, Jan K. Buitelaar, Daniel Brandeis, Tobias Banaschewski, Anna Kaiser, Sarah Hohmann, Bob Oranje, Bram Gooskens, Sarah Durston, Steven C.R. Williams, David J. Lythgoe, Jilly Naaijen

**Affiliations:** a Department of Cognitive Neuroscience, Donders Institute of Brain, Cognition and Behaviour, Radboud University Medical Center, the Netherlands; b Karakter Child and Adolescent Psychiatry University Center, Nijmegen, the Netherlands; c Department of Child and Adolescent Psychiatry and Psychotherapy, Central Institute of Mental Health, Medical Faculty, Mannheim/Heidelberg University, Mannheim, Germany; d Department of Child and Adolescent Psychiatry and Psychotherapy, Psychiatric Hospital, University of Zurich, Zurich, Switzerland; e Center for Integrative Human Physiology, University of Zurich, Zurich, Switzerland; f Neuroscience Center Zurich, University of Zurich, Zurich, Switzerland; g ETH Zurich, Zurich, Switzerland; h Department of Psychiatry, Brain Center, University Medical Center Utrecht, Utrecht University, Utrecht, the Netherlands; i Department of Neuroimaging, King’s College London, Institute of Psychiatry, Psychology and Neuroscience, London, United Kingdom; j Donders Centre for Cognitive Neuroimaging, Donders Institute for Brain, Cognition and Behaviour, Radboud University, the Netherlands

**Keywords:** Compulsivity, Fronto-striatal, Glutamate, Spectroscopy, Inhibitory control

## Abstract

Autism spectrum disorder (ASD) and obsessive compulsive disorder (OCD) show overlapping symptomatology and deficits in inhibitory control, which are associated with altered functioning and glutamatergic signaling in fronto-striatal circuitry. These parameters have never been examined together. The purpose of the current study was to investigate functioning during inhibitory control and its association with fronto-striatal glutamate concentrations across these disorders using a multi-center, longitudinal approach. Adolescents with ASD (n = 24), OCD (n = 15) and controls (n = 35) underwent two magnetic resonance imaging (MRI) sessions with a one-year interval. This included proton magnetic resonance spectroscopy (^1^H-MRS; n = 74) and functional MRI during an inhibitory control task (n = 53). We investigated ^1^H-MRS data and fMRI data separately as well as integrated in a multimodal analysis using linear models focusing on diagnosis and continuous measures of overlapping compulsivity symptoms. ACC glutamate was reduced over time in the ASD group compared with controls, while striatal glutamate decreased over time independent of diagnosis. Increased compulsive behavior seemed to be associated with increased striatal activity during failed inhibitory control. The integrated analyses showed differential involvement of increased striatal glutamate during failed but decreased striatal glutamate during successful inhibitory control in the OCD group compared to controls and ASD, suggesting different underlying mechanisms for OCD compared to ASD.

## Highlights

• Multi-center, longitudinal, transdiagnostic study of glutamate and neural activity.
• Differing roles of glutamate on activity in striatum during inhibitory control.
• Glutamate concentrations in ACC decrease over time in ASD adolescents.
• Differing neural mechanisms of compulsivity in OCD and repetitive behaviors in ASD.

## 1 Introduction

Although autism spectrum disorder (ASD) and obsessive compulsive disorder (OCD) are two separate neurodevelopmental disorders with distinct diagnostic characteristics (American Psychiatric Association, 2013), they are highly comorbid and a comparison of symptoms has suggested more similarities than differences between the two (Robbins et al., 2012, Anholt et al., 2010, Zandt et al., 2007). However, not much is known about underlying mechanisms of the behaviors common among those with these disorders; restricted and repetitive patterns of behavior and/or compulsivity. The latter is defined as a repetitive, irresistible urge to perform certain behaviors or thoughts, and diminished control over this urge (Chamberlain and Menzies, 2009). Repetitive and compulsive behaviors are associated with deficits in inhibitory control in tasks such as the stop-signal task (Anholt et al., 2010, Chamberlain et al., 2007). Fronto-striatal areas are known to be involved in inhibitory control, and are regulated by the excitatory neurotransmitter glutamate (Naaijen et al., 2015, Naaijen et al., 2017, Naaijen et al., 2018). Within fronto-striatal circuity, the striatum is thought to be involved in driving the repetitive and compulsive behaviors, while frontal regions, such as the anterior cingulate cortex (ACC) is responsible for exerting inhibitory control (Naaijen et al., 2015, Bari and Robbins, 2013, Fineberg et al., 2010, Aron et al., 2007, Aron and Poldrack, 2006, Dalley et al., 2011, Zandbelt et al., 2010). Imaging studies focusing on ASD and/or OCD have shown altered fronto-striatal structure and function as well as altered glutamate conentrations, suggesting a possible shared underlying mechanism affecting repetitive and compulsive behaviors (Bari and Robbins, 2013, Fineberg et al., 2010, Chmielewski and Beste, 2015). Here, we investigated this by using a multi-center, longitudinal approach looking at associations of fronto-striatal glutamate and repetitive and compulsive behaviors on neural activity during inhibitory control in a childhood/adolescent cross-disorder population.

In studies using the stop-signal task in ASD and OCD, there have been inconsistent results. Some studies found no behavioral differences in ASD and OCD compared with controls (Chantiluke et al., 2015, Albajara Sáenz et al., 2020, Gooskens et al., 2018), while others have found worse performance in participants with OCD (Chamberlain and Menzies, 2009, Chamberlain et al., 2007, Verbruggen and Logan, 2008, de Wit et al., 2012, Penadés et al., 2007, Norman et al., 2019), demonstrating deficits in inhibitory control. However, these differences are more commonly found in adults with OCD than children and adolescents (Marzuki et al., 2020). Altered activity in fronto-striatal areas during inhibitory control has been found in both disorders as well (Albajara Sáenz et al., 2020, Norman et al., 2019, Apergis-Schoute et al., 2018, Woolley et al., 2008, Carlisi et al., 2017), showing reduced activity during inhibition in ACC. Additionally, in ASD increased activation has been found in left striatum compared to controls, while this was decreased in OCD (Carlisi et al., 2017). Contrarily, some studies found altered functional activity despite not finding behavioral differences in response inhibition compared to controls (Roth et al., 2007, Rubia et al., 2010). In a previous study using a partly overlapping sample of the current study, no behavioral or neural alterations were found during inhibitory control in participants with ASD and OCD (Gooskens et al., 2018).

The excitatory neurotransmitter glutamate is highly relevant for proper fronto-striatal functioning as well as inhibitory control. Altered concentrations of glutamate, investigated using proton magnetic resonance spectroscopy (^1^H-MRS), have been linked to repetitive behaviors and compulsivity (Naaijen et al., 2015, Silverman et al., 2008), which seem to differ in individuals with ASD and OCD compared to controls across development. A meta-analysis of ^1^H-MRS studies investigating fronto-striatal glutamate in the neurodevelopmental disorders ASD, OCD and attention-deficit/hyperactivity disorder (ADHD) reported that increased glutamate concentrations in striatum seems to be present across these disorders (Naaijen et al., 2015). In the ACC, on the other hand, glutamate concentrations were often higher in children and adolescents with these disorders while in adults the opposite pattern was found, with lower concentrations compared to controls, suggesting a developmental shift (Naaijen et al., 2015). In a study investigating glutamate concentrations and neural functioning during inhibitory control, increased ACC glutamate was associated with decreased activity in striatum, but this was unrelated to any clinical diagnosis (Naaijen et al., 2018).

Evidence from these previous studies strongly suggests that investigating the interplay between glutamate and functional activity during inhibitory control is an important step for understanding the mechanistic underpinnings of behaviors across neurodevelopmental disorders. In a study including the first time of measure of the participants in this study, increased ACC glutamate was found in both ASD and OCD, and a positive association between ACC glutamate and compulsive behaviors was found, while there were no group differences in striatal glutamate nor any association with behavior (Naaijen et al., 2017). In the current study we followed up this sample with a second timepoint of measurements using a multimodal, multi-center study design. With this developmental data we aimed to investigate whether changes in fronto-striatal glutamatergic alterations and functioning during inhibitory control differed across (atypical) neurodevelopment and whether there were any changes over time. Based on previous findings, we expect increased glutamate concentrations in fronto-striatal brain regions in the ASD and OCD group, especially in the ACC. As repetitive and compulsive behaviors likely decrease over time, we expect inhibitory control to be associated with these behaviors differently over time. In addition, we expect a differential role for glutamate here, which may affect functioning differently in ASD and OCD as compared to the control group. These are exploratory analyses as the link between fronto-striatal functioning and neurochemistry has not been investigated in these groups before.

## 2 Methods and materials

### 2.1 Participants

We included 74 participants (ASD = 24, OCD = 15, controls = 35) for the ^1^H-MRS analysis, between 8 and 16 years old at time-point 1 (T1), and between 9 and 17 years at timepoint 2 (T2). Our previous manuscript describing the spectroscopy results of T1 included a total amount of n = 133 participants (Naaijen et al., 2017). Reasons for drop-out for this longitudinal study were loss of interest by the participants and quality restrictions regarding the spectra. For the combined ^1^H-MRS and fMRI analysis we included 53 participants. The participants were recruited at three different locations across Europe (Radboud University Medical Center and the Donders Institute for Brain, Cognition and Behavior, Nijmegen, The Netherlands (N = 38), Kings College London, London, United Kingdom (N = 17), Central Institute of Mental Health, Mannheim, Germany (N = 19)) in the multicenter study COMPULS, part of the TACTICS consortium (www.tactics-project.eu). Another site was excluded due to too few participants surviving quality control (N = 3). The inclusion criteria were IQ > 70, ability to speak and comprehend the native language of the location of recruitment and being of Caucasian descent (for further details, see (Naaijen et al., 2017). To confirm DSM-IV-TR (Association, 2000) diagnoses of ASD and OCD, we used the Autism Diagnostic Interview-Revised (ADI-R) (Lord et al., 1994) and Childreńs Yale Brown Obsessive Compulsive Scale (CYBOCS) (Scahill et al., 1997) for ASD and OCD respectively. Participants with ASD and OCD were not allowed to have a diagnosis of the other disorder of interest. Control participants were confirmed to not score in the clinical range for any DSM IV axis I diagnoses using the Child Behavior Checklist (CBCL) and the Teacher Report Form (TRF) (Bordin et al., 2013), assessment of ADHD symptoms were measured using the Conners Parent Rating Scale (CPRS-R, (Conners et al., 1998). Repetitive and compulsive behaviors were measured using the Repetitive Behavior Scale – Revised (RBS-R) (Lam and Aman, 2007). Information on medication use was collected on the measurement days via parental report. Participants were asked to abstain from stimulant medication 48 h before scanning. None of the participants received non-pharmacological treatment during the study. Ethical approval for the study was obtained for all centers separately and participants and their parents gave written informed consent for participation.

### 2.2 Stop-signal task

To measure inhibitory control participants completed a modified visual version of the stop-signal task (SST) (Rubia et al., 2003) during an fMRI session. For details of the design of the task see Fig. S1 in the Supplementary material. To ensure consistency across sites, task instructions were given according to a standard operating procedure (SOP).

### 2.3 Image acquisition

Participants were familiarized with the MRI settings and practiced the SST using a dummy scanner at T1. At T2, the task was practiced again if needed. The data were acquired from the three study locations, all using 3 Tesla scanners (Siemens Trio and Siemens Prisma, Siemens, Erlangen, Germany; Philips Achieva, Philips Medical Systems, Best, The Netherlands; General Electric MR750, GE Medical Systems, Milwaukee, Wi, USA). Structural T1-weighted scans were acquired based on the ADNI GO protocols (Jack et al., 2008, Jack et al., 2010), which were used for registration of the functional scans and voxel placement for the ^1^H-MRS.

Spectra were acquired using a point resolved spectroscopy sequence (PRESS) with a chemically selective water suppression (CHESS) (Haase et al., 1985) from the midline pregenual ACC and the left dorsal striatum covering caudate and putamen with an 8 cm^3^ voxel size (2 × 2 × 2). Voxel locations were adjusted to maximize the amount of grey matter (GM) and minimize the cerebrospinal fluid (CSF) content to keep the quality of the data as high as possible. The locations of all voxel placements are shown in the Supplementary material (Fig. S2 and S3). Details on the structural, functional and ^1^H-MRS scan parameters can be found in Table S1 in the Supplementary material.

### 2.4 Imaging analysis

#### 2.4.1 fMRI

From the 74 participants included in analysis based on available MRS data, 53 had available fMRI data included in analysis (ACC: ASD = 15, OCD = 11, Controls = 27; Striatum: ASD = 13, OCD = 9, Controls, 24). Preprocessing of the fMRI data was performed using FSL (https://fsl.fmrib.ox.ac.uk/fsl/). The first five volumes from each scan were removed to account for equilibration effects. Head movement correction was performed by realigning to the middle volume (MCFLIRT; (Jenkinson et al., 2002)). A Gaussian kernel with full width at half maximum (FWHM) of 6 mm was used for grand mean scaling and spatial smoothing. ICA-AROMA (Pruim et al., 2015a, Pruim et al., 2015b) was then used to remove signal components related to secondary-head motion artefacts, subsequently followed by nuisance regression to remove signal from CSF and white matter (WM), and high-pass filtering (100sec). These images were co-registered to each participants’ anatomical scan using boundary-based registration within FSL-FLIRT (Greve and Fischl, 2009). The anatomical scans were spatially normalized using a 12-parameter affine registration to MNI152 standard space, which was refined by non-linear registration FSL-FNIRT (Andersson et al., 2007). The images were then brought into standard space by applying the resulting warp fields to the concatenated functional image. Neural activation during inhibitory control was analyzed using SPM12 (Statistical Parametric Mapping release 12, https://www.fil.ion.ucl.ac.uk/spm/). For the whole-brain analysis during the stop-task, the first level models included two contrasts of interest; (American Psychiatric Association, 2013) failed stop – successful go, to isolate failed inhibitory control and (Robbins et al., 2012) successful stop – failed stop, to isolate successful inhibitory control. For the second level analyses regarding differences across groups and times of measure, we used a full-factorial design where t-contrasts were applied to the first level contrast maps. To investigate the association between our spectral data and the fMRI data we extracted the mean beta weights during both failed and successful inhibitory control from the ACC and dorsal striatum regions of interest as extracted from the ^1^H-MRS voxels. This was done using the MarsBar toolbox (Brett et al., 2002).

#### 2.4.2 ^1^H-Mrs

**Fig. 1:**
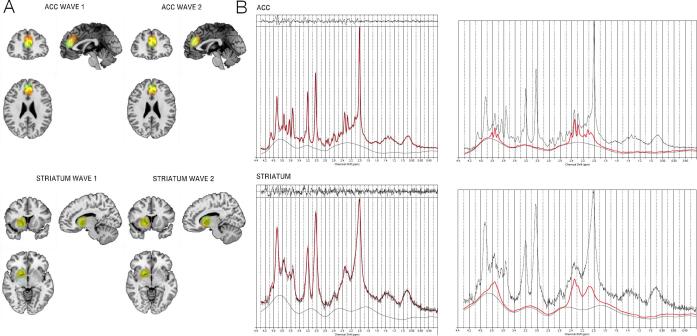
^1^H-MRS voxel placement. A: Superposition on the MNI152 template of all individual voxel placements in ACC and striatum, for ASD (red), OCD (blue) and controls (yellow). The placements were consistent across diagnoses, as seen by the large overlap of voxels. For voxel placements across sites, see Supplementary material. B: Example spectra of a 3 T proton magnetic resonance spectroscopy (^1^H-MRS) Linear Combination (LC) Model spectral fit in ACC and striatum from one of the control participants. The top of the images represents the residuals. The black line represents frequency-domain data, the red line is the LCModel fit. The right images show the fits for glutamate only. For examples of LCModel spectral fits and glutamate fits for each site, see Fig. S5 in the Supplementary material. (For interpretation of the references to colour in this figure legend, the reader is referred to the web version of this article.)

Glutamate concentrations were estimated using Linear Combination Model (LCModel), using water as reference (Provencher, 2014, Provencher, 2001). Example fitted spectra for ACC and striatum can be seen in Fig. 1. As different tissues contain different amounts of water, correction for tissue percentage and partial volume effects was calculated using the formula:

$${\mathrm{Metabolite}}_{\mathrm{corrected}}={\mathrm{Metabolite}}_{\mathrm{Raw}}\times \left(\frac{\left(43300\times f_{\mathrm{GM}}+35880\times f_{\mathrm{WM}}+55556\times f_{\mathrm{CSF}}\right)}{35880}\right)\times \left(\frac{1}{\left(1-f_{\mathrm{CSF}}\right)}\right)$$

where 43 300 is the water concentration in millimolar for GM, 35 880 for WM and 55 556 for CSF, as described in the LCModel manual (Provencher, 2014).

Criteria for quality control were the signal-to-noise ratio being ≥ 15, Cramér-Rao lower bounds ≤ 20%, and FWHM ≤ 0.1 parts per million. This resulted in 74 participants included in the analysis of ACC glutamate (ASD = 24, OCD = 15, Controls = 35), and 55 participants included for striatal glutamate (ASD = 18, OCD = 11, Controls = 26). To check for possible influences of glutamine we performed quality controls of glutamine concentrations, which only survived quality control measures for one participant for the ACC voxel and ten participants for the striatum voxel. We therefore do not report Glx (glutamate + glutamine) measures and report only glutamate. The raw glutamate levels can be found in Table S3 in the Supplementary material.

### 2.5 Statistical analyses

Statistical analyses were performed using the R-software package (Booth et al., 2018), unless otherwise stated.

We first investigated changes in fronto-striatal glutamate concentrations, neural activation and behavioral responses during inhibitory control over time separately. Changes in these scores over time were calculated by subtracting glutamate levels, or neural responses, in the spectral regions of interest and measures of compulsivity and inhibitory control at T1 from T2. These are reported as change-scores (Δ). Diagnosis, Δ RBS-R total and Δ RBS-R compulsivity scores were then used as predictors in separate models. Age, sex and scan-site were included as covariates of non-interest in all analyses; because age and sex did not affect the results nor influenced the model(s), they were removed from further analyses. To test general effects of time we used linear mixed effects models, where participant was added as a random factor to account for within subject variability across time (lme4 package (Bates et al., 2015)). Additionally, we investigated whether ADHD symptoms was associated with glutamate concentrations by including the CPRS-R scores in separate models. As there were no associations of ADHD symptoms, CPRS-R scores were not included in subsequent models in analyses.

Secondly, we combined spectral and functional analyses into a multi-modal model investigating whether changes over time in one modality were associated with changes over time in the other modality using the ^1^H-MRS voxels as regions of interest. Specifically, we investigated whether changes in glutamate concentrations in either region (ΔGluACC/Str) were associated with changes in neural activation (ΔbetaACC/Str) in the same region and whether this was different across groups and continuous measures of repetitive behavior. This resulted in twenty-four models, which are shown in more detail in Table S2 in the Supplementary material.

All reported *p*-values in all statistical tests are corrected for multiple comparisons by the false discovery rate (FDR, *q* < 0.05), unless otherwise stated. Effect sizes are indicated as *r*.

## 3 Results

### 3.1 Demographics

**Fig. 2:**
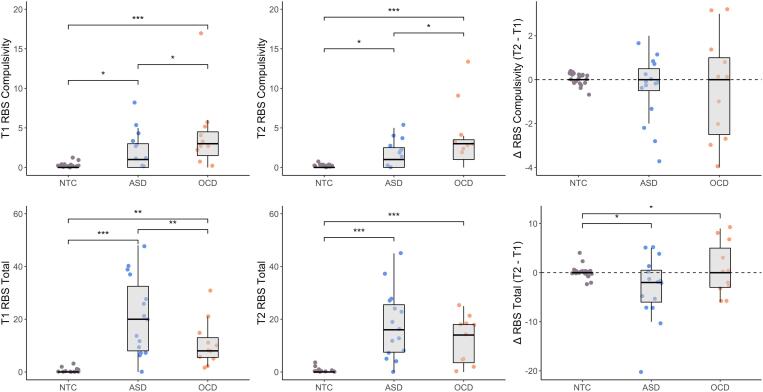
Repetitive and compulsive behaviors. Group differences in RBS compulsivity (upper panel) and RBS total scores (lower panel) at T1, T2 and over time. The OCD (N = 15) group showed higher compulsivity than both ASD (N = 24) and controls (N = 35) at both time-points without any differences in changes. Total RBS scores were highest in the ASD group at both time-points while simultaneously they showed the largest decrease in these symptoms between T2 and T1. * *p* < 0.05, ** p < 0.01, *** *p* < 0.001.

No differences were found between groups in age, IQ or sex. Table 1 shows an overview of the demographics and clinical variables of the largest subsample used. For the repetitive and compulsive behaviors we used the RBS-R total scores and the compulsivity subscale scores at T1, T2 as well as the change over time (Δ). Although there was no general effect of time on these measures, there were significant differences between ASD, OCD, and controls at all time-points. See Fig. 2 for a summary of these results.

**Table 1:** Demographic characteristics (based on the largest subsample group in analysis).

	ASD		OCD		Controls		Test statistic	p-value	Post-hoc
Sex, m/f	17/7		9/6		21/14		KWχ_2_ = 0.81	0.667	
	Mean	SD	Mean	SD	Mean	SD			
Age 1	11.38	1.64	11.95	2.49	10.70	1.38	KWχ_2_ = 5.61	0.061	
Age 2	12.92	1.62	13.38	2.51	12.20	1.46	KWχ_2_ = 5.27	0.072	
IQ^a^	109.38	15.07	109.89	15.56	111.84	11.05	KWχ_2_ = 0.50	0.781	

RBS 1									
Total	24.86	24.46	15.67	19.09	0.80	2.14	KWχ_2_ = 49.75	<0.001	OCD & ASD > Controls
Stereotype	2.79	3.27	2.00	2.80	0.06	0.24	KWχ_2_ = 31.44	<0.001	OCD & ASD > Controls
Self-harm	1.38	2.06	1.40	2.77	0.06	0.34	KWχ_2_ = 20.18	<0.001	OCD & ASD > Controls
Compulsivity	3.46	5.74	4.73	5.00	0.20	0.63	KWχ_2_ = 29.12	<0.001	OCD & ASD > Controls
Ritualistic	5.17	6.03	3.73	4.30	0.08	0.28	KWχ_2_ = 40.04	<0.001	OCD & ASD > Controls
Insist on sameness	9.71	8.61	2.87	5.89	0.26	0.92	KWχ_2_ = 42.22	<0.001	ASD > OCD & Controls
Limited interests	2.46	2.78	0.93	1.16	0.14	0.36	KWχ_2_ = 20.42	<0.001	ASD & OCD > Controls

RBS 2									
Total	20.61	19.48	11.86	9.71	0.46	1.06	KWχ_2_ = 44.26	<0.001	OCD & ASD > Controls
Stereotype	2.26	2.25	1.71	1.69	0.03	0.17	KWχ_2_ = 31.99	<0.001	OCD & ASD > Controls
Self-harm	1.96	3.62	0.71	1.69	0.00	0.00	KWχ_2_ = 16.68	<0.001	ASD > OCD & Controls
Compulsivity	2.43	3.46	3.71	3.73	0.06	0.24	KWχ_2_ = 30.08	<0.001	OCD & ASD > Controls
Ritualistic	3.83	4.31	2.29	2.91	0.12	0.33	KWχ_2_ = 20.17	<0.001	OCD & ASD > Controls
Insist on sameness	7.63	6.34	2.07	2.62	0.18	0.53	KWχ_2_ = 37.76	<0.001	OCD > ASD > Controls
Limited interests	2.30	2.75	1.36	1.45	0.06	0.24	KWχ_2_ = 24.63	<0.001	OCD & ASD > Controls

MEDICATION^b^									
Stimulant	2		0		0				
Antipsychotic	0		1		0				
Antidepressant	1		5		0				

ASD, Autism Spectrum Disorder; OCD, Obsessive Compulsive Disorder; SD, standard deviation; RBS, Repetitive Behavior Scale (Lam and Aman, 2007). KWχ^2^; Kruskal-Wallis Chi-Square. Post hoc tests were Bonferroni corrected. The number of participants per group is the largest subsample available across analyses. a IQ was collected during the first time of measure. b Medication use is indicated from first time of measure, changes in the second measure can be found in the supplementary material. 1 and 2 in the left column indicate first (T1) and second (T2) point of measure.

### 3.2 Spectral quality

Groups did not differ in mean voxel percentage GM, WM or CSF in both voxels (all *p*-values > 0.05). Percentage GM in striatum, however, was lower the second time of measure compared to the first one ((b = −0.07, *t*_(52)_ = −2.97, *p* = 0.004), independent of diagnosis. Voxel placement during T1 and T2 and across scan-sites can be seen in the Supplementary material in Figs. S2 and S3. No differences were found between diagnostic groups or time-points for any of the measures. The ASD group showed, compared with controls, an increase in glutamate Cramér-Rao lower bound (CRLB) over time (b = 0.009, *t*_(71)_ = 2.49, *p* = 0.015), although with the highest CRLB of 14%, guaranteeing sufficient quality of these spectra at both timepoints (Kreis, 2016).

### 3.3 Fronto-striatal glutamate

**Fig. 3:**
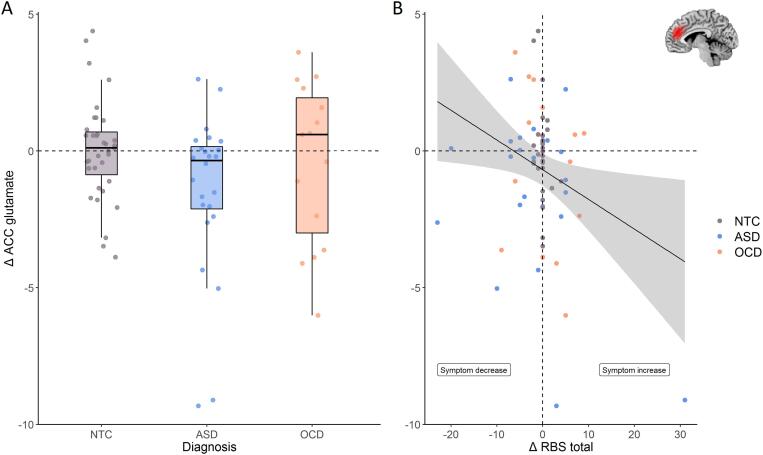
ACC glutamate. A: Glutamate concentrations, shown in institutional units (i.u.), decreased over time in the ASD (N = 24) group (blue) compared with controls (N = 35) (grey). Plot was created using ggplot2 (Ginestet, 2011) and in-house adapted violin plots (Hintze and Nelson, 1998). * *p* < 0.05. B: Effects of changes of changes in RBS-R total score on changes in ACC glutamate (in i.u.). The linear regression line shows a negative association of Δ RBS-R total score with changes Δ ACC glutamate, independent of diagnosis. The shaded area represents the 95% confidence interval. Dots on the vertical dashed line represent participants that did not change in RBS-R total scores. *Note*: this figure shows raw data-points, not model estimates. (For interpretation of the references to colour in this figure legend, the reader is referred to the web version of this article.)

There was a negative association between diagnosis and ΔGluACC (b = −1.55, t=_(0.68)_ = −2.28, *p* = 0.026, *r* = 1.00), which indicated a larger decrease in ACC glutamate in ASD over time compared with controls, but not OCD (*p* > 0.05; Fig. 3A). In addition, the RBS-R total score was associated with ACC glutamate as well, where an increase over time in repetitive behaviors was related to a decrease over time in ACC glutamate (b = −0.12, t_(0.05)_ = −2.330, *p* = 0.026, *r* = 1.00; Fig. 3B).

There was no effect of diagnostic status or any of the continuous measures on ΔGluStr (all *p*-values > 0.05). However, striatal glutamate decreased significantly over time, independent of diagnosis (b = −0.65, t_(52)_ = −2.77, *p* = 0.023, *r* = 0.36).

### 3.4 Stop-signal task

All groups showed common patterns of brain activation during failed as well as successful inhibitory control, where there was activation in areas typically associated with inhibitory control, such as ACC and striatum (Fig. S4). No significant differences in neural activation between groups were found at any time-point in any of our contrasts (all *p*-values > 0.05). However, using continuous measures of compulsivity and our fronto-striatal regions of interest, we found an effect of Δ compulsivity on Δ striatal activity (b = 1.88, t_(0.51)_ = 3.70, *p* = 0.002, *r* = 0.98) during failed inhibitory control, where an increase in compulsivity over time was associated with an increased striatal activation, reflecting higher activity at T2, compared to T1. Behavioral results regarding the SST are described further in the supplementary material.

### 3.5 Association between fronto-striatal glutamate and functioning

#### 3.5.1 Failed inhibitory control

**Fig. 4:**
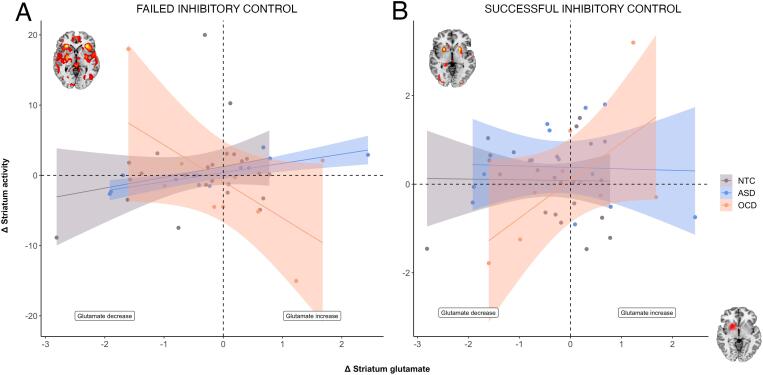
Failed and Successful inhibitory control. A: During failed inhibitory control, an increase in striatal glutamate (i.u.) was associated with a decrease in striatal BOLD signal in the OCD (N = 9) group (salmon) compared to controls (N = 24) (grey) and ASD (N = 13) (blue). B: During successful inhibitory control, an increase in striatal glutamate (i.u.) was associated with an increase in striatal BOLD in the OCD group compared to controls and ASD. Brain activity is shown on the axial slice for both failed and successful inhibitory control outlining the striatal voxel as an overlay. Activity is presented at *p* < 0.01 (uncorrected) for visualization purposes. The shaded areas represent the 95% confidence intervals. *Note:* This figure shows raw data-points, not model estimates. (For interpretation of the references to colour in this figure legend, the reader is referred to the web version of this article.)

During failed inhibitory control there was a negative interaction between diagnosis and ΔGluStr on ΔbetaStr. This interaction showed that in OCD, an increase in striatal glutamate over time was associated with a decrease over time in activity in the same region compared to controls (b = −7.46, t_(2.19)_ = −3.412, *p* = 0.003, *r* = 0.92), and ASD (b = 7.73, t_(2.30)_ = 3.36, *p* = 0.003, *r* = 0.91); see Fig. 4A. There was no significant difference between ASD and controls (all *p*-values > 0.05). No associations were found regarding the ACC or any interactions between glutamatergic changes and continuous measures of compulsivity (all *p*-values > 0.05).

#### 3.5.2 Successful inhibitory control

During successful inhibitory control, there was a positive interaction between diagnosis and ΔGluStr on ΔbetaStr. This time, again in OCD, an increase in striatal glutamate over time was associated with an increase in striatal activity control compared to controls (b = 0.96, t_(0.41)_ = 2.33, *p* = 0.025, *r* = 0.96), and ASD (b = 1.04, t_(0.43)_ = 2.40, *p* = 0.025, *r* = 0.96), see Fig. 4B. There was again no significant difference between ASD and controls (all *p*-values > 0.05) nor any other significant associations for the ACC or continuous measures of compulsivity (all *p*-values > 0.05).

## 4 Discussion

This is the first study that used a multi-center, longitudinal, transdiagnostic approach to investigate the associations of repetitive behaviors and compulsivity with fronto-striatal glutamate concentrations and functioning during inhibitory control in a childhood/adolescent cross-disorder population.

Our ^1^H-MRS only results showed that over time there was a reduction in ACC glutamate in the ASD group compared with controls, while an increase in repetitive behaviors over time was associated with decreased glutamate in the same region. Previous studies investigating children with ASD have shown higher glutamate concentrations in ACC (Bejjani et al., 2012, Hassan et al., 2013, Joshi et al., 2012), while studies looking at adults with ASD have found both lower and higher glutamate concentrations in ACC compared to controls (Naaijen et al., 2015, Ford and Crewther, 2016). Our finding may therefore reflect changes in development in ASD being different from development in controls. We found no such differences in the OCD group, although they did not significantly differ from the ASD group either, and previous studies with OCD have shown inconsistent results (Naaijen et al., 2015). This may be due to a larger heterogeneity in the disorder, and future studies considering possible subtypes of OCD may successfully disentangle such differing results. However, the previous study investigating an overlapping sample (however, larger) at T1 found increased ACC glutamate in both ASD and OCD (Naaijen et al., 2017). In the striatum we found that glutamate decreased over time independent of diagnosis. This is in line with the study that found no group differences in striatal glutamate during the first time of measure (Naaijen et al., 2017), which is reflected also at T2. Alterations in metabolite concentrations during development are also known to occur in controls (Horská et al., 2002), and our finding may reflect such development in striatum, independent of a clinical diagnosis.

Regarding neural activation, we did not find any group differences, time effects nor effects of our continuous measures in our whole brain analyses for neither failed nor successful inhibitory control. This was in line with the findings of T1 by Gooskens et al. (2018). However, other studies with similar behavioral results still found altered brain activation during inhibitory control (Chantiluke et al., 2015, Norman et al., 2019, Woolley et al., 2008, Carlisi et al., 2017, Rubia et al., 2010). Although we were not able to find any whole-brain differences, when we were looking at our region of interest, we found that an increase in compulsivity over time was associated with increased striatal activation over time, but only during failed inhibitory control. Increased compulsivity may thus be associated with more difficulties with inhibition, resulting in more striatal activity reflecting an increased cognitive demand. Our longitudinal TACTICS study on inhibitory control in ASD and OCD found improvements in SSRT over time, regardless of diagnosis (Gooskens et al., 2018). In our partly overlapping subsample in this study, as shown in the supplementary material, we do not replicate this finding but show that males performed better than females. The fact that we did not find a general improvement may, however, be due to a lack of power and/or a larger proportion of males in this subsample.

Integrating all these analyses for the first time in a multi-modal fashion investigating the association between developmental changes in glutamate concentrations as well as fronto-striatal functioning resulted in differential findings across failed and successful inhibitory control. While during failed inhibitory control, OCD participants showed decreased striatal activity with an increase in striatal glutamate over time, the reverse was found for successful inhibitory control; increased concentrations were associated with increased activity, again in the OCD group. Both these findings were significant compared with controls as well as compared with ASD. These results suggest differential involvement of striatal glutamate in neural activation patterns in OCD compared with controls and ASD during different aspects of inhibitory control. In order to successfully inhibit responses, more glutamate resulted in more activity, suggesting a compensatory mechanism in order to fulfill the cognitive demands of the task, even though behaviorally there were no differences in performance. As these results show significant changes over time in our ~1 year time window between measurements, our results also suggest there may be critical differences in neural measurements in childhood/adolescent neurodevelopmental populations. This needs further investigation, but may explain inconsistent results in neuroimaging results with child/adolescent populations in these disorders.

Considering that the OCD group showed higher compulsivity scores compared to controls as well as compared to ASD without any changes over time (Fig. 2), associations of both changes of glutamate in OCD and compulsivity on striatal activity during failed inhibitory control may point towards the same mechanistic differences for achieving the same neural activation. A recent study using a network analysis has suggested that compulsivity as seen in OCD and repetitive behaviors as seen in ASD represent distinct features of these disorders (Ruzzano et al., 2015), rather than symptom overlap between the two; something that has also been suggested (Ivarsson and Melin, 2008, Bartz and Hollander, 2006). Our OCD and ASD results do not overlap, but were found within the different regions of the fronto-striatal circuit (OCD findings in the striatum, ASD findings in the ACC). This indeed suggests that compulsivity in OCD and repetitive behaviors in ASD have distinct mechanistic underpinnings that are regionally specific and differently regulated by glutamate, despite the seemingly similar behavioral phenotypes. Considering the very limited research on these measures during adolescence, even more so in OCD than in ASD, these results are an important step towards increasing understanding of underlying mechanisms of development in compulsivity-related disorders. Further studies should confirm this initial finding, but this may contribute to targeted glutamate altering interventions in OCD.

Strengths of the current study are combining categorical and dimensional analyses, with a longitudinal approach to investigate the relationship between repetitive and compulsive behaviors, fronto-striatal glutamate as well as functioning. There were also some limitations. Firstly, the OCD group was smaller than the ASD group, which may have led to less power and the possibility of false negatives. However, we still found significant associations with changes in glutamate concentrations affecting changes in functional activity in OCD. Furthermore, the percentage GM in striatum decreased over time, suggesting worse voxel placement. However, these changes were not different across diagnostic groups and therefore probably did not affect our main findings. As ability to speak their native language and IQ > 70 were inclusion criteria in this study, this may have resulted in excluding what is often considered “low functioning” ASD participants. Therefore, our ASD specific results may not be generalizable to the entire population of individuals with ASD. There are also difficulties performing multicenter studies, where data quality may differ across sites. However, we did manage to control for these effects in our models and our results were likely not affected by left-over site effects. Future studies should use a true longitudinal model with a longer time-period in between as well as preferably a larger sample size to increase the understanding of these integrated mechanisms underlying ASD and OCD. To further investigate similarities and differences between these disorders regarding compulsivity and repetitive behaviors we also suggest using a larger battery of measures of compulsivity and repetitive behaviors, to disentangle what variations of these features differ between these diagnostic groups, and what their underlying mechanisms are.

## 5 Conclusion

In conclusion we found, over time, significant associations in OCD of increased glutamate concentrations in striatum with decreased functional activity in striatum during failed inhibitory control, and an opposite effect of increased striatal glutamate concentrations with increased striatal activity during successful inhibitory control. Increased compulsivity was also associated with increased striatal activity during failed inhibitory control. While glutamatergic alterations were differently involved during neural activation in OCD, there were no general changes in glutamate in the OCD group over time compared with controls. In ASD on the other hand, we found ACC glutamate to decrease more over time compared with controls. These results should be replicated in an independent sample, but this study has given new insights into the alterations of glutamate in ASD and OCD during development in adolescence, and its role in functional activity.

## CRediT authorship contribution statement

**Viola Hollestein:** Formal analysis, Software, Data curation, Writing - original draft, Writing - review & editing, Visualization. **Jan K. Buitelaar:** Conceptualization, Validation, Project administration, Funding acquisition. **Daniel Brandeis:** Conceptualization, Methodology, Writing - review & editing. **Tobias Banaschewski:** Conceptualization, Methodology, Writing - review & editing. **Anna Kaiser:** Writing - review & editing. **Sarah Hohmann:** Writing - review & editing. **Bob Oranje:** Conceptualization, Methodology, Writing - review & editing. **Bram Gooskens:** Conceptualization, Methodology, Writing - review & editing. **Sarah Durston:** Conceptualization, Methodology, Writing - review & editing. **Steven C.R. Williams:** Conceptualization, Methodology, Writing - review & editing. **David J. Lythgoe:** Conceptualization, Methodology, Writing - review & editing. **Jilly Naaijen:** Conceptualization, Methodology, Software, Investigation, Data curation, Writing - review & editing, Supervision, Project administration.

## Declaration of Competing Interest

JK Buitelaar has been consultant to/member of advisory board of and/or speaker for Janssen Cilag BV, Eli Lilly, Bristol- Myer Squibb, Shering Plough, UCB, Shire, Novartis, and Servier. He is not an employee of any of these companies, nor a stock shareholder of any of these companies. He has no other financial or material support, including expert testimony, patents, and royalties. D Brandeis serves as an unpaid scientific advisor for an EU-funded Neurofeedback trial unrelated to the present work. T Banaschewski served in an advisory or consultancy role for Actelion, Hexal Pharma, Lilly, Medice, Novartis, Oxford outcomes, PCM scientific, Shire, and Viforpharma. He received conference support or speaker’s fee by Medice, Novartis, and Shire. He is/has been involved in clinical trials conducted by Shire and Viforpharma. DJ Lythgoe has acted as a consultant for Ixico PLC. The remaining authors declare no conflict of interest. The present work is unrelated to the grants and relationships noted earlier.
