# Supplementary material for: Developmental changes in fronto-striatal glutamate and their association with functioning during inhibitory control in autism spectrum disorder and obsessive compulsive disorder
Source: Neuroimage Clin. 2021 Mar 10;30:102622. doi: 10.1016/j.nicl.2021.102622 (PMC8022251; doi:10.1016/j.nicl.2021.102622)
Supplement: Supplementary data 1 [file mmc1.docx]

# **Supplementary information**

**Developmental changes in fronto-striatal glutamate and their association with functioning during inhibitory control in autism spectrum disorder and obsessive compulsive disorder**

**Hollestein et al.**


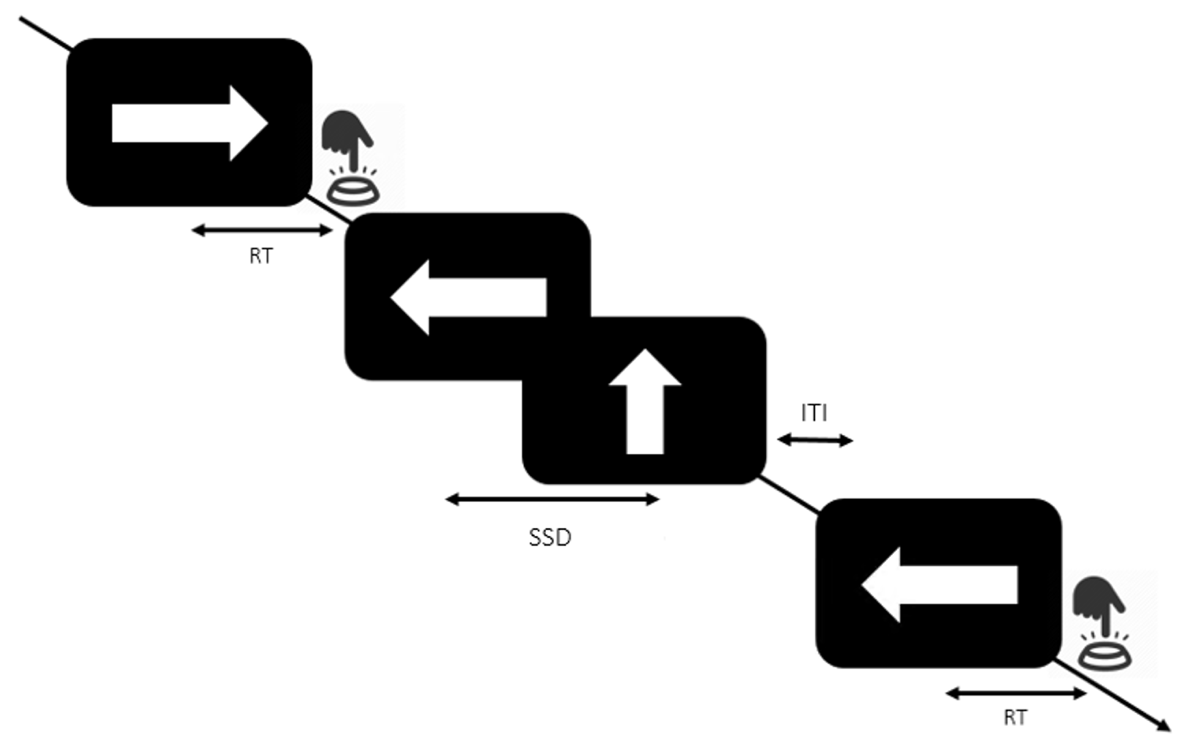


**Figure S1:** Stop-signal task. Arrows were presented on a screen; the task was to press a button indicating the direction the arrow was pointing at. In 20% of trials the arrow was followed by a stop cue of an arrow pointing upwards, instructing to withhold a response. The stop-signal delay (SSD) between stimulus onset and stop-signal was adaptive, where the SSD after successful inhibition increased with 50 ms while after failed inhibition it decreased with 50 ms. This ensured participants success to inhibit in approximately 50% of the stop-trials. The inter-trial interval (ITI), the time between the trials, was randomly jittered between 1.6 and 2.0 seconds.


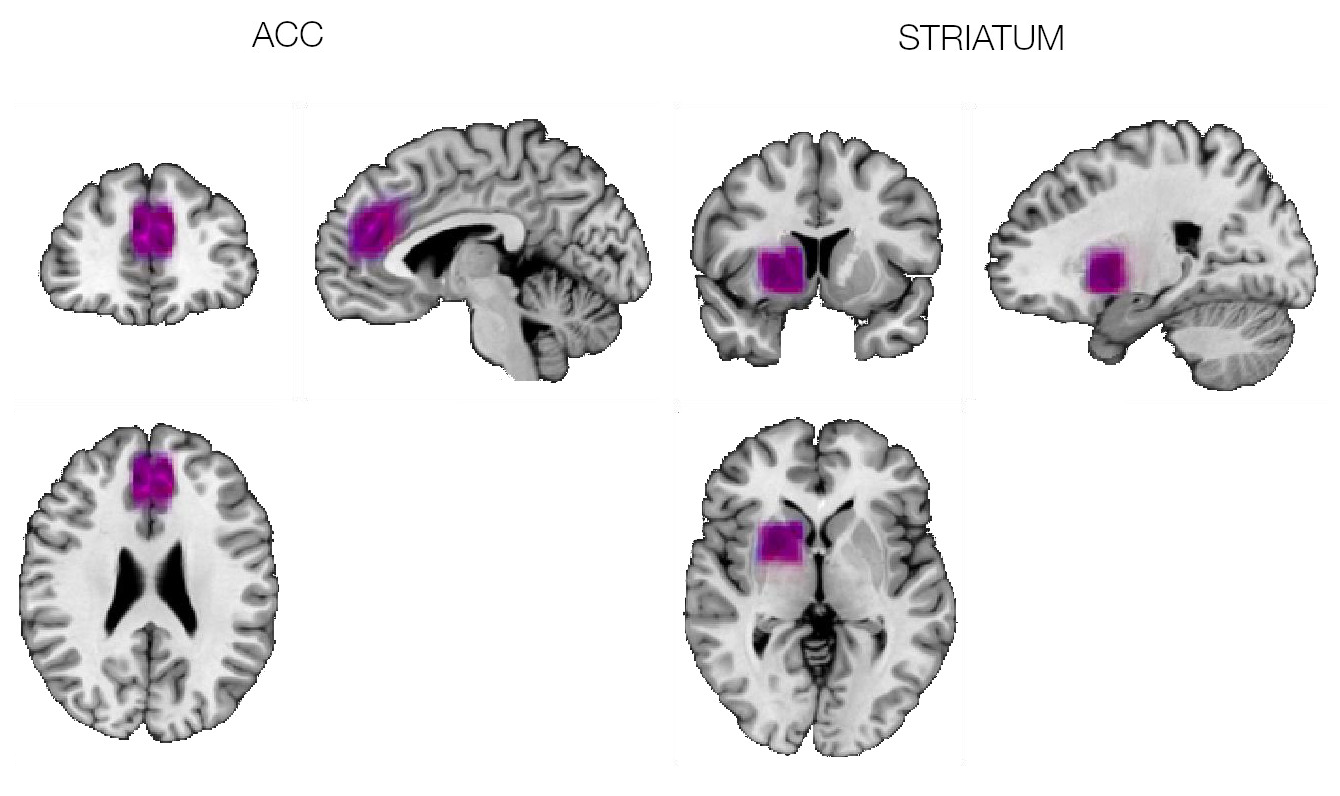


**Figure S2:** Superposition on the MNI152 template of all individual voxel placements in ACC (left) and striatum (right), across times (First time of measure, blue; Second time of measure, red). The placements are consistent across times of measures.


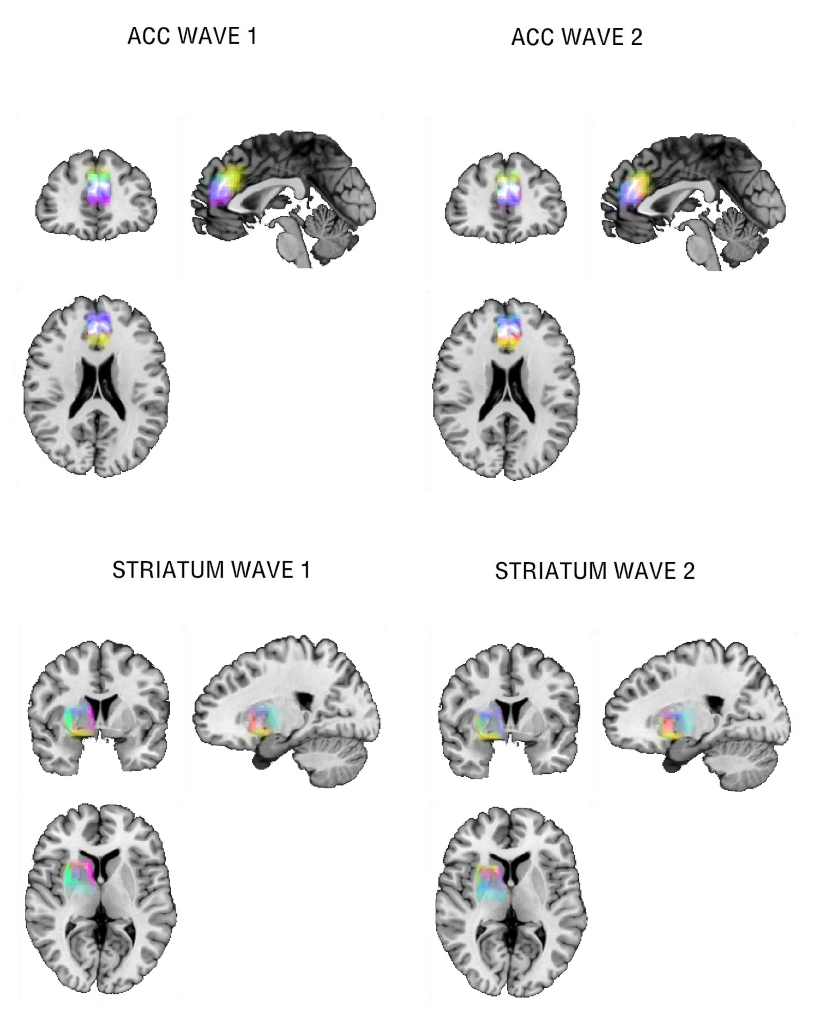


**Figure S3:** Superposition on the MNI152 template of all individual voxel placements in ACC and striatum, for all sites (London, blue; Mannheim, yellow; Nijmegen, pink). The placements are consistent across and within sites. For more detail across-site acquisition, see (1) and (2).


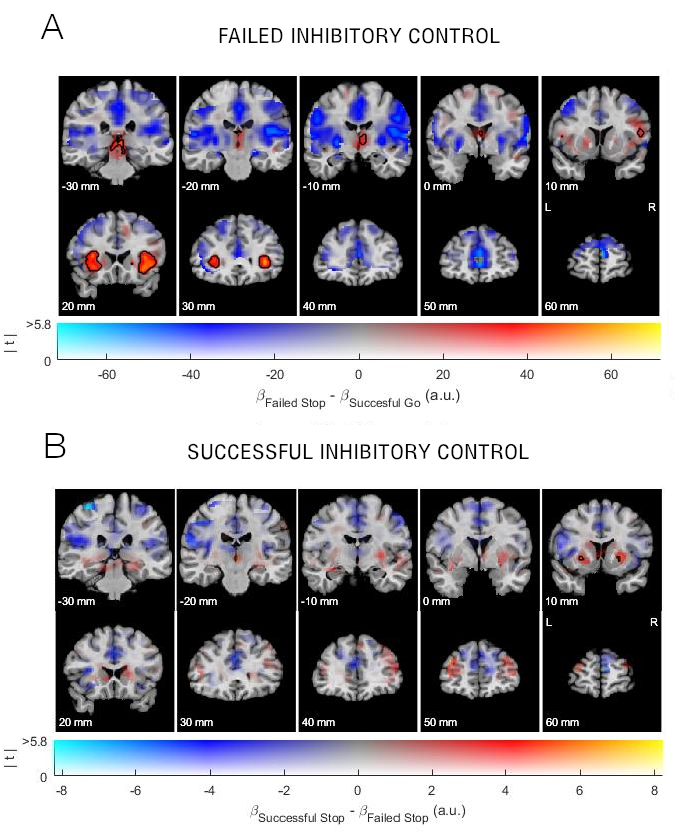


**Figure S4:** Task activation across all groups during (A) failed inhibitory control (failed stop – successful go) and (B) successful inhibitory control (successful stop – failed stop), which showed common patterns of activation. The colors reflect uncorrected activation, voxels with a black line around the color reflect survived correction at *p*_FWE_ = 0.05 showing fronto-striatal activation during cognitive control. The numbers below the color bars reflect beta-values. Neuroimaging data are plotted using a procedure introduced by (3) and implemented by (4).


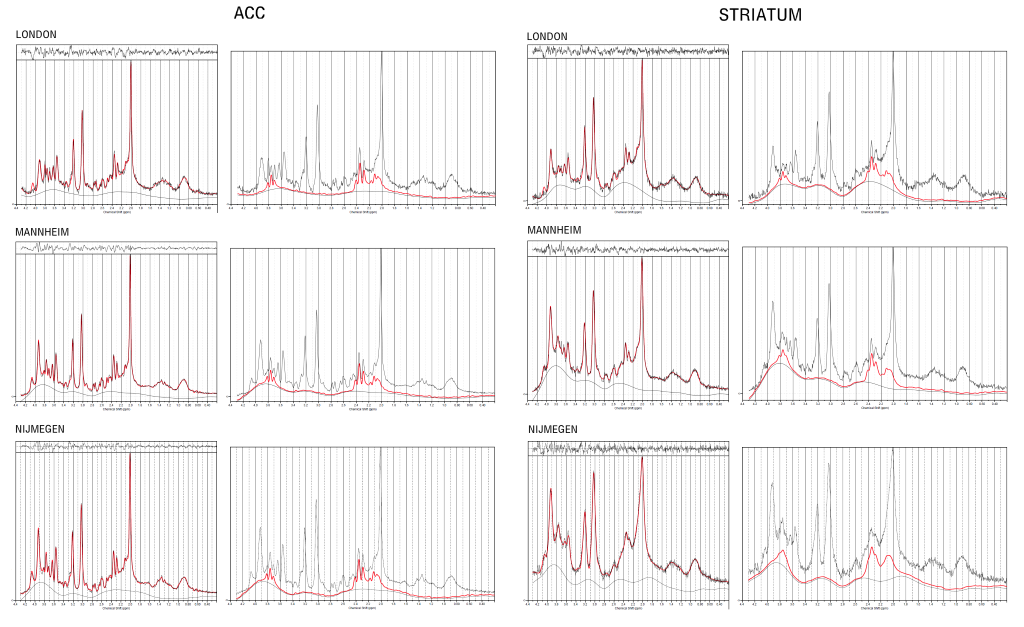


**Figure S5:** Example spectra of a 3T from proton magnetic resonance spectroscopy (^1^H-MRS) Linear Combination (LC) Model spectral fit in ACC and striatum across all sites from separate participants. The top of the images represents the residuals. The black line represents frequency-domain data, the red line is the LCModel fit. The right images show the fits for glutamate only.

**Table S1:** Scan sequences

| Sequence | Site | TR/TE/TI (ms) | Flip angle | Field of  view (mm) | Matrix RL/AP/slices | Voxel – size (mm) | Gap (%) | Parallel Imaging | Averages Water  suppressed/  unsuppressed |
| --- | --- | --- | --- | --- | --- | --- | --- | --- | --- |
| T1 | Nijmegen  (Siemens) | 2300^*^/2.98/900 | 9 | 256 | 212/256/176 | 1.0´1.0´1.2 | NA | 2 | NA |
|  | Mannheim  (Siemens) | 2300^*^/2.98/900 | 9 | 270 | 212/254/176 | 1.1´1.1´1.2 | NA | 2 | NA |
|  | London  (GE) | 7.31^*^/3.02/400 | 11 | 270 | 256/256/196 | 1.1´1.1´1.2 | NA | 1.75 | NA |
| ^1^H-MRS PRESS | All | 3000/30/- | NA | NA | NA | 20´20´20 | NA | NA | 96/16 |
| Functional MRI | All | 2070/35/- | 74 | 192 | 192/192/36 | 3.0´3.0´3.0 | 13 | 2 | NA |

*As provided by the manufacturer. GE defines a TR as the time between excitation pulses, while Siemens defines TR as the time between inversion recovery pulses.

**Details multimodal analysis**

Linear models were used for our multimodal statistical analyses using the lm function available in the base package in R (RStudio Team, 2016). Our integrated analyses of the ^1^H-MRS and fMRI data resulted in twenty-four models: Δbeta in ACC or striatum during failed or successful inhibitory control were dependent variables (4), and our (continuous) predictors of interest were ΔGluACC or ΔGluStr (2), together with diagnostic status, ΔRBS compulsivity or ΔRBS total (3); 4 x 2 x 3 = 24 models. Site was added as a predictor of non-interest to all models, to account for site-effects on our measures. All models are listed in Table S2. These models test associations of the predictors (right side of Table S2) on neural activity in our regions of interest during inhibitory control (left side of Table S2).

**Table S2:** Linear regression models of multimodal analyses

| **Failed inhibitory control** |  |
| --- | --- |
| ΔbetaACC | ~ ΔGluACC * Diagnosis + Site |
| ΔbetaACC | ~ ΔGluACC * Δ RBS Total score + Site |
| ΔbetaACC | ~ ΔGluACC * Δ RBS Compulsivity + Site |
| ΔbetaACC | ~ ΔGluStr * Diagnosis + Site |
| ΔbetaACC | ~ ΔGluStr * Δ RBS Total score + Site |
| ΔbetaACC | ~ ΔGluStr * Δ RBS Compulsivity + Site |
| ΔbetaStr | ~ ΔGluACC * Diagnosis + Site |
| ΔbetaStr | ~ ΔGluACC * Δ RBS Total score + Site |
| ΔbetaStr | ~ ΔGluACC * Δ RBS Compulsivity + Site |
| ΔbetaStr | ~ ΔGluStr * Diagnosis + Site |
| ΔbetaStr | ~ ΔGluStr * Δ RBS Total score + Site |
| ΔbetaStr | ~ ΔGluStr * Δ RBS Compulsivity + Site |
| **Successful inhibitory control** |  |
| ΔbetaACC | ~ ΔGluACC * Diagnosis + Site |
| ΔbetaACC | ~ ΔGluACC * Δ RBS Total score + Site |
| ΔbetaACC | ~ ΔGluACC * Δ RBS Compulsivity + Site |
| ΔbetaACC | ~ ΔGluStr * Diagnosis + Site |
| ΔbetaACC | ~ ΔGluStr * Δ RBS Total score + Site |
| ΔbetaACC | ~ ΔGluStr * Δ RBS Compulsivity + Site |
| ΔbetaStr | ~ ΔGluACC * Diagnosis + Site |
| ΔbetaStr | ~ ΔGluACC * Δ RBS Total score + Site |
| ΔbetaStr | ~ ΔGluACC * Δ RBS Compulsivity + Site |
| ΔbetaStr | ~ ΔGluStr * Diagnosis + Site |
| ΔbetaStr | ~ ΔGluStr * Δ RBS Total score + Site |
| ΔbetaStr | ~ ΔGluStr * Δ RBS Compulsivity + Site |

ΔbetaACC/ ΔbetaStr: Changes in neural activation in ACC/striatum between time-point 1 (T1) and time-point 2 (T2), during failed or successful inhibitory control. ΔGluACC/ ΔGluAStr: Changes in glutamate concentration in ACC/striatum between T1 and T2. “~” indicates that the variables on the right side are associated with the dependent variable on the left hand side. The “x” between the variables of interest indicated that the model assess these variables both independently and their interaction effects.

For analyses of glutamate concentrations in ACC and striatum associated with time and diagnosis independently, linear mixed effects models were used using the lme4 package (Bates et al., 2014). The lmer function was used to fit linear mixed-effects models:

GluACC ~ Diagnosis * Time + Site + (1|Participant)

GluStr ~ Diagnosis * Time + Site + (1|Participant)

For analysis of SSRT group comparison over time the following model was used:

SSRT ~ Diagnosis * Time + Site + (1|Participant)

The linear mixed effects models account for within subject variability over time by adding participant as a random factor.

**Table S3:** Raw glutamate levels at T1 and T2

| Diagnosis | ACC T1 | ACC T2 | STR T1 | STR T2 |
| --- | --- | --- | --- | --- |
| ASD | 10.46646461 | 8.496578355 | NA | NA |
| ASD | 9.502124709 | 9.887580258 | 8.247075833 | 6.352032402 |
| ASD | 10.45382499 | 8.428490578 | NA | NA |
| ASD | 9.808432659 | 9.344632423 | 6.673729796 | 6.263571408 |
| ASD | 9.03451682 | 9.839876294 | 6.941402246 | 6.487048865 |
| ASD | 8.459706588 | 8.431155876 | 6.536547776 | 4.619588719 |
| ASD | 10.12216162 | 10.4795116 | 4.877940024 | 7.320578073 |
| ASD | 12.28622171 | 7.932510748 | 4.56654398 | 5.242980373 |
| ASD | 9.528864663 | 9.623110444 | 5.694679044 | 6.126748945 |
| ASD | 8.762677237 | 7.701688908 | 7.093335165 | 6.294975144 |
| ASD | 8.678187241 | 9.172212197 | NA | NA |
| ASD | 15.74052401 | 15.49530981 | 9.466125109 | 9.237021295 |
| ASD | 22.9679727 | 13.85815441 | 9.251698141 | 11.4537308 |
| ASD | 16.79127614 | 14.39287469 | 8.051919207 | 9.004611253 |
| ASD | 14.73175341 | 16.9883202 | 9.28496324 | 9.112406219 |
| ASD | 13.63378153 | 16.26286471 | 9.060665591 | 7.549782644 |
| ASD | 14.17480125 | 12.66238483 | 9.056145735 | 7.362930309 |
| ASD | 12.12576328 | 11.93199961 | NA | NA |
| ASD | 9.161339697 | 9.192108149 | NA | NA |
| ASD | 10.09289446 | 8.424581493 | NA | NA |
| ASD | 10.61616663 | 10.41175931 | 6.46558221 | 7.254520547 |
| ASD | 10.9101927 | 8.294028346 | 6.704347808 | 5.705254053 |
| ASD | 20.07059274 | 15.04236284 | 6.313574257 | 6.402479415 |
| ASD | 17.61304837 | 8.295779564 | 6.927463488 | 6.594419822 |
| Controls | 9.099615118 | 8.483204361 | 7.037234954 | 5.448861084 |
| Controls | 11.90266353 | 10.1138775 | 8.379938904 | 7.000655466 |
| Controls | 9.703567829 | 9.071750281 | 7.80662892 | 6.190655013 |
| Controls | 9.390472411 | 9.032679628 | 6.445081954 | 6.760872956 |
| Controls | 9.688986736 | 8.583364547 | NA | NA |
| Controls | 11.67860923 | 14.28663776 | NA | NA |
| Controls | 12.75080489 | 9.575479647 | 6.614020154 | 6.303383585 |
| Controls | 7.664403144 | 7.655782073 | 8.646897829 | 5.834470139 |
| Controls | 9.424915728 | 9.03764206 | 6.412028842 | 7.0184551 |
| Controls | 11.35464345 | 9.890793357 | 7.099357725 | 7.158730934 |
| Controls | 14.18055305 | 10.30268859 | 6.500372186 | 6.313729084 |
| Controls | 8.3599096 | 9.949890644 | 6.344679076 | 6.160679051 |
| Controls | 9.675412031 | 9.555373079 | NA | NA |
| Controls | 9.532707858 | 8.17309906 | 5.971965993 | 6.647455325 |
| Controls | 9.554553965 | 10.11461868 | 6.188594005 | 5.432526183 |
| Controls | 8.349128716 | 9.130057346 | NA | NA |
| Controls | 8.845944014 | 9.417837617 | 6.252027577 | 5.607146096 |
| Controls | 9.468649279 | 9.049949317 | 4.859555094 | 5.479611342 |
| Controls | 14.53116037 | 11.055685 | 9.642807214 | 9.761815574 |
| Controls | 11.92673935 | 15.96033783 | 11.03101319 | 7.788142954 |
| Controls | 15.70143114 | 18.91421894 | 7.214572233 | 6.967431191 |
| Controls | 15.02416225 | 13.30618856 | 8.520991737 | 8.595460476 |
| Controls | 13.61447189 | 14.73748597 | 9.814362703 | 5.817394356 |
| Controls | 10.8603263 | 10.68386152 | NA | NA |
| Controls | 10.38257723 | 10.58082587 | NA | NA |
| Controls | 11.79813299 | 13.02120024 | NA | NA |
| Controls | 14.25759296 | 18.65058519 | 7.763861404 | 8.162968401 |
| Controls | 9.722633896 | 10.94151912 | 7.176305053 | 7.359308648 |
| Controls | 10.54027896 | 11.11133059 | 7.462122855 | 6.350588479 |
| Controls | 9.969426778 | 10.08564778 | 7.538577217 | 5.966455811 |
| Controls | 9.988677383 | 10.25062889 | 7.257006015 | 8.034547418 |
| Controls | 10.55478596 | 10.94177261 | NA | NA |
| Controls | 9.578996296 | 9.882392411 | NA | NA |
| Controls | 13.57801178 | 11.51251984 | 7.014844338 | 6.712492963 |
| Controls | 10.50094717 | 11.10952951 | 6.496388591 | 5.760879882 |
| OCD | 13.53502686 | 9.430361242 | 5.592711434 | 4.605072501 |
| OCD | 9.947264788 | 10.54916246 | 7.556859004 | 5.955580429 |
| OCD | 10.08391323 | 10.7309773 | 6.900305508 | 7.096163611 |
| OCD | 10.67196045 | 13.28778394 | 7.404535854 | 6.701631818 |
| OCD | 14.57241252 | 10.94868352 | 6.65173351 | 6.705290913 |
| OCD | 9.625646412 | 9.238435885 | 5.329838352 | 5.325569179 |
| OCD | 9.316942712 | 10.35378667 | 5.431188384 | 6.662102284 |
| OCD | 10.74705388 | 8.375389987 | NA | NA |
| OCD | 8.194909742 | 11.80746102 | 6.14599681 | 6.142299143 |
| OCD | 17.78596703 | 13.89954418 | 10.13001278 | 11.80747576 |
| OCD | 23.88093187 | 17.86656965 | NA | NA |
| OCD | 12.65959818 | 14.95887336 | NA | NA |
| OCD | 14.9685669 | 17.69010235 | 7.334637431 | 7.923531508 |
| OCD | 13.88118108 | 15.47390753 | NA | NA |
| OCD | 10.6982443 | 9.595557239 | 6.996366196 | 6.843144398 |
|  |  |  |  |  |

**Medication use over time**

During the first time of measure, in the ASD group two people used stimulants, and one anti-depressants. In the OCD group five people used antidepressants and one anti-psychotics. In the second time of measure, one of the participants using stimulants and the participant using anti-depressants now also used antipsychotics. An additional participant used antidepressants, and one antipsychotics and stimulants. In the OCD group two were no longer on antidepressants, the one using antipsychotics in the first time of measure now also used antidepressants, and one participant had started using stimulants. None of the controls used medication at any time of measure.

**Stop-Signal task (behavioral)**

**Analysis.** The behavioral measure of interest on the SST was the stop-signal reaction time (SSRT), which was calculated using the integration method (5,6), where the reaction time (RT) of correct go trials was rank ordered, then the *n*th go-RT was selected, where *n* was derived by multiplying the number of correct go-trials by the probability that the participant respond to a stop signal. The SSRT was then estimated by subtracting the mean SSD from the *n*th go-RT (7). Participants were excluded from analysis for excessive motion or when they showed an SSRT < 50 ms as it is indicative of not performing the task properly, for example by constantly pressing buttons without paying attention to cues which results in atypically short response times on correct go-trials. This resulted in 41 participants included for stop-task analysis (ASD = 12, OCD = 8, controls = 21). Data from T1 and T2 were initially analyzed separately allowing investigation of group differences without the possible influence of time. Shapiro-Wilk normality tests showed that there was no normal distribution in neither time of measure, and therefore Fligner-Killeen tests of homogeneity of variance were used, which showed that there was equal variance between diagnosis groups in both T1 and T2. Consequently, Kruskal-Wallis tests were used to analyze differences in SSRT between groups (ASD, OCD or controls) for T1 and T2 independently. To investigate behavioral differences between groups on the stop-task measured by the SSRT, Kruskal-Wallis tests were used to compare groups, and a mixed effects model was used to analyze changes between groups over time of measure, including the same covariates as described before.

**Results.** There were no group differences in SSRT at T1 (c^2^_(2)_= 2.84, *p* > 0.1) or T2(c^2^_(2)_=2.64, *p* > 0.1), showing similar performance across groups. Across T1 and T2 a significant effect of sex was found (b = -101.34, t_(34.3)_=-2.21 *p* = 0.03, *r* = 0.35), indicating an improvement in stop-task performance in males, but not in females. There were also no significant group differences in task performance across T1 and T2.

**Supplemental references**

1. Naaijen J, de Ruiter S, Zwiers MP, Glennon JC, Durston S, Lythgoe DJ, et al. COMPULS: Design of a multicenter phenotypic, cognitive, genetic, and magnetic resonance imaging study in children with compulsive syndromes. BMC Psychiatry. 2016;16(1):1–10.

2. Naaijen J, Zwiers MP, Amiri H, Williams SCR, Durston S, Oranje B, et al. Fronto-striatal glutamate in autism spectrum disorder and obsessive compulsive disorder. Neuropsychopharmacology. 2017;42(12):2456–65.

3. Allen EA, Erhardt EB, Calhoun VD. NeuroView Data Visualization in the Neurosciences : Overcoming the Curse of Dimensionality NeuroView. Neuron. 2012;74(4):603–8.

4. B Z. Slice Display figure. Figshare. 10.6084/m9.figshare.4742866. 2017.

5. Verbruggen F, Aron AR, Band GPH, Beste C, Bissett PG, Brockett AT, et al. A consensus guide to capturing the ability to inhibit actions and impulsive behaviors in the stop-signal task. eLife. 2019;

6. Verbruggen F, Chambers CD, Logan GD. Fictitious Inhibitory Differences: How Skewness and Slowing Distort the Estimation of Stopping Latencies. Psychological Science. 2013;

7. Verbruggen F, Logan GD. Response inhibition in the stop-signal paradigm. Trends in Cognitive Sciences. 2008.
